# The role of coronary artery collaterals in the preservation of left ventricular function: a study to address a longstanding controversy

**DOI:** 10.5830/CVJA-2016-054

**Published:** 2017

**Authors:** NO Ajayi, KS Satyapal, EA Vanker

**Affiliations:** Department of Clinical Anatomy, School of Laboratory Medicine and Medical Sciences, College of Health Sciences, University of KwaZulu-Natal, Westville, Durban, South Africa; Department of Clinical Anatomy, School of Laboratory Medicine and Medical Sciences, College of Health Sciences, University of KwaZulu-Natal, Westville, Durban, South Africa; St Augustine’s Hospital, Chelmsford Medical Centre, Durban, South Africa

**Keywords:** coronary artery obstruction, coronary collateral artery, ventricular function

## Abstract

**Introduction:**

The functional significance of coronary artery collateral (CAC) vasculature in humans has been debated for decades and this has been compounded by the lack of a standard, systematic, objective method of grading and documenting CAC flow in man. CACs serve as alternative conduits for blood in obstructive coronary artery disease. This study aimed to evaluate the impact of CACs on left ventricular function in the presence of total coronary arterial occlusion.

**Methods:**

The study group included the coronary angiographic records of 97 patients (mean age: 59 ± 8 years). CACs were graded from 0–3 based on the collateral connection between the donor and recipient arteries. Left ventricular function was computed from the ventriculogram and expressed as ejection fraction (EF).

**Results:**

The mean EF of the patients with grades 0, 1, 2 and 3 CACs were calculated as 50.4, 47, 60.5 and 70%, respectively. A significant difference was recorded in the mean EF calculated for the different CAC grades (p = 0.001). There was a significant positive correlation (p < 0.001; r = 0.478) between the mean EF and the CAC grades.

**Conclusion:**

The patients with better coronary collateral grades had a higher mean EF. Therefore, as the grade of CACs increased, there was an improvement in their ability to preserve left ventricular function.

## Introduction

Controversy has existed for decades regarding the functional significance of coronary artery collaterals (CACs) in humans,^1^ and this has been compounded by the lack of a standard systematic method for determining CAC flow in man.^2^,^3^ These CACs are reported to have a protective effect on myocardial perfusion and contractile function, and to prevent left ventricular (LV) aneurysm formation in the presence of severe coronary artery obstruction.^4^,^5^ The presence of collateral vessels may play a major role in determining whether the patient will develop symptoms of myocardial ischaemia and vulnerability of the myocardium to myocardial infarction.^6^

The use of coronary angiography allows correlation of the extent of development of CACs with the severity of coronary arterial disease.^7^,^8^ The presence of functional collateral vessels can be of important prognostic value and can also assist in determining the need for intervention and the type of interventional procedure to be performed.^9^,^10^ Indeed, the presence of well-developed coronary collateral vasculature and flow has been correlated with the absence of ischaemic symptoms in patients with established coronary artery disease (CAD).^11^

Meier and colleagues,^12^ in an analysis of previous studies on the effect of coronary collaterals on mortality, reported that well-developed collaterals reduced the mortality rate in the order of 35%. The presence of adequately developed collateral supply limits the degree of myocardial necrosis during myocardial infarction.^8^,^13^ The area at risk of myocardial infarction is inversely related to the collateral supply to that region, and therefore becomes zero in the presence of well-developed functional collaterals.^14^-^16^ In cases of unsuccessful intra-coronary thrombolytic therapy after the onset of symptoms in acute myocardial infarction, the improvement in LV function and wall motion in the infarct region have been associated with the presence of collateral flow to the region perfused by the obstructed vessel.^5^,^17^

However, some reports have cast doubt on the value of CACs. Banerjee reported that the presence of CACs had no protective role on the incidence of LV aneurysm formation following myocardial infarction.^18^ Ilia et al.^19^ also reported that there was no correlation between the characteristics of CACs and the presence or absence of LV systolic abnormality in patients with significant CAD. Furthermore, Turgut et al.^20^ stated that coronary collaterals did not have a protective role on preservation of LV function in the presence of severe left anterior descending artery stenosis. Meier et al. also reported that the development of good CACs increased the risk of restenosis after percutaneous coronary intervention.^21^ In view of these controversies, our study was undertaken to evaluate the effect of CACs on LV function in the setting of a totally occluded coronary artery demonstrated on angiogram.

## Methods

The study group was selected from the reviewed angiographic records of 2 029 consecutive patients (mean age: 59 ± 12 years) who had had coronary catheterisation performed by interventional cardiologists for symptoms suggestive of CAD. In order to assess the effect of CACs on LV function in the presence of total occlusion of the coronary artery, only those coronary angiograms that had LV function assessed by ventriculography were selected for analysis.

Ninety-seven such patients with total occlusion of a coronary artery and LV functional assessment were included in the analysed angiograms. The mean EF and the different grades of CACs in these patients were determined.

The angiograms were obtained from the cardiac catheterisation laboratories of hospitals within the private sector in the eThekwini municipality region of KwaZulu-Natal, South Africa. Ethical approval (ethics number BE 196/13) for the study was obtained from the University of KwaZulu-Natal Biomedical Research Ethics Committee.

Coronary arteriography was performed via the percutaneous transfemoral approach by injecting a radio-opaque contrast agent into the coronary blood vessels, and the images were taken using X-ray fluoroscopy. These images were recorded on digital media in DICOM (Digital Imaging and Communication in Medicine) format and stored in the cardiac catheterisation laboratories.

The relationship between the location of the atherosclerotic lesions and the CAC grades were examined in the angiograms that met the inclusion criteria. In addition, the relationship between the location of the atherosclerotic lesions and the mean EF was also evaluated. The location of atherosclerotic lesion was determined by dividing the coronary arteries into proximal, middle and distal regions.

The Rentrop grading system22 is the most widely used grading system for coronary collaterals and is employed by many researchers. However, most patients are graded Rentrop 2 or 3 in chronic total coronary occlusion.^23^

**Fig. 1.**
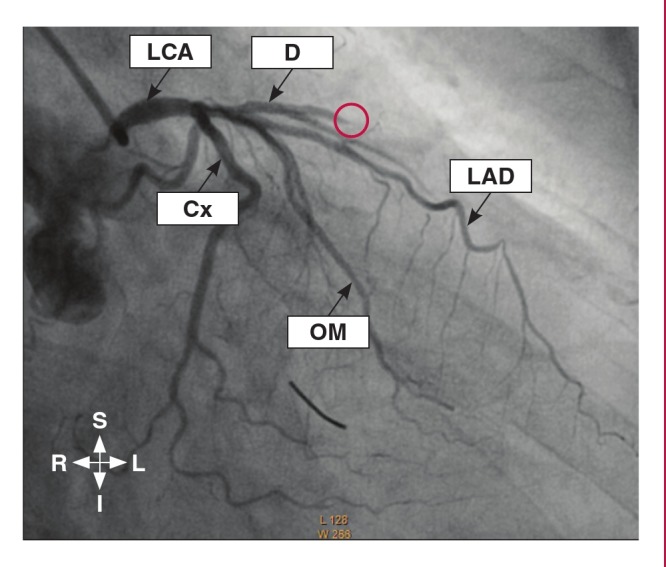
Coronary angiogram in the right anterior oblique view (caudal angulation) showing obstruction of the diagonal branch of the left anterior descending (LAD) artery (red ring) without collateral vessels to the distal segment of the obstructed vessel. LCA, left coronary artery; D, diagonal; Cx, circumflex; OM, obtuse marginal artery.

**Fig. 2.**
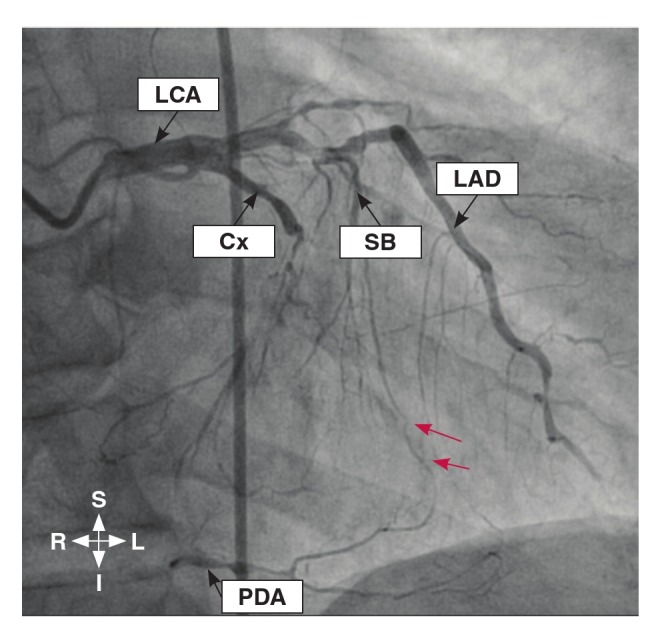
Coronary angiogram in the right anterior oblique view showing the filling of the posterior descending artery of an obstructed right coronary artery (RCA) by grade 1 collateral vessel (red arrows) originating from the septal branch of the left anterior descending (LAD) artery. LCA, left coronary artery; Cx, circumflex; SB, septal branch; PDA, posterior descending artery.

**Fig. 3.**
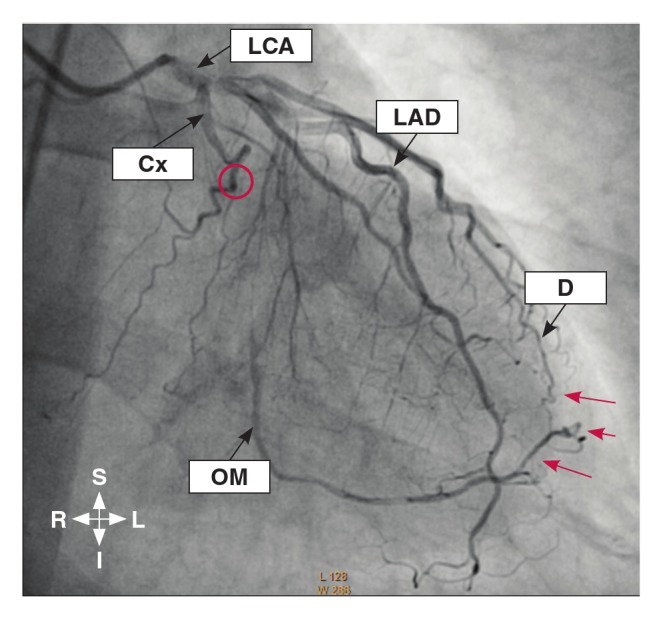
Coronary angiogram in the right anterior oblique view (caudal angulation) showing obstruction of the circumflex (Cx) branch (red ring) with the filling of the obtuse marginal (OM) branch by grade 3 collateral vessel (red arrows) originating from the diagonal branch of the left anterior descending (LAD) artery. D, diagonal.

The grading of the coronary collaterals in the present study was based on the grading system used by Werner et al.,^24^ with the addition of a grade for absent CACs. This system centered on defining the collateral connection between the donor and the recipient arteries. Therefore, in this study, the coronary collaterals were graded as: grade 0 for absent collateralisation, where there were no demonstrable CACs to the distal region of the obstructed vessel (Fig. 1); grade 1 for poor collateralisation, where there were CACs showing no continuous connection between the donor and recipient arteries (Fig. 2); grade 2 for good collateralisation, where there were continuous threadlike connections between the donor and recipient arteries; and grade 3 for excellent collateralisation, where there were continuous prominent connections with side branches between the donor and recipient arteries (Fig. 3).

Data were analysed with the Statistical Package for the Social Sciences (SPSS) version 21 for Windows (IBM SPSS, NY, USA). A p-value < 0.05 was considered statistically significant.

## Results

The mean age of the patients with coronary artery occlusion who had LV function assessed by ventriculography was 59 ± 8 years. The patients consisted of 25.8% females and 74.2% males (Table 1). The grades of the CACs were as follows: absent (15.4%), poor (15.4%), good (36.9%) and excellent (32.3%). The morphological properties of the coronary arterial tree in the analysed angiograms are shown in Table 1.

**Table 1:** Parameters of patients who had left ventriculography performed

Parameters	Values (n = 97)
Mean age, years (SD)	59.1 (8.8)
Mean ejection fraction, % (SD)	60.2 (18.1)
Gender (%)	
Female	25.8
Male	74.2
Coronary dominance (%)	
Co-dominance	3.1
Left	13.4
Right	83.5
Location of obstruction (%)	
Proximal	45.4
Middle	38.1
Distal	16.5
Grading of collateral (%)	
Absent	15.4
Poor	15.4
Good	36.9
Excellent	32.3

The grades of the collateral pathways with regard to the location of atherosclerotic obstruction were evaluated. They were recorded as 15.9, 9.1, 34.1 and 40.9% in the proximal region of the coronary arteries for absent, poor, good and excellent CACs, respectively. The grades of the collateral pathways with obstruction of the middle region were recorded as 16.2, 16.2, 37.8 and 29.7% for absent, poor, good and excellent CACs, respectively. The grades of the collateral pathways with the obstruction of the distal region were recorded as 18.8, 18.8, 37.5 and 25% for absent, poor, good and excellent CACs, respectively. There was no significant difference in the grades of CACs between the different regions of obstruction (p = 0.87) (2Table 2).

**Table 2:** Grading of coronary collateral pathways in the obstruction of the different regions of the main coronary arteries in patients who had left ventriculography

Obstructed coronary arterial region	Grades of collateral vessel (%)				
	Absent	Poor	Good	Excellent	p-value
Proximal	15.9	9.1	34.1	40.9	0.87
Middle	16.2	16.2	37.8	29.7	
Distal	18.8	18.8	37.5	25	

The mean EF of the patients with proximal, middle and distal location of atherosclerotic lesions was 63.3, 57.8 and 57.5%, respectively. This indicated that the best mean EF was recorded in the patients with proximally located atherosclerotic lesions. However, analysis of variance (ANOVA) showed that there was no significant difference in the mean EF calculated for the different locations of atherosclerotic lesions (p = 0.33) (Table 3).

**Table 3:** Mean ejection fraction of patients in the different locations of obstructive atherosclerotic lesions

Lesion location	Sample size (n)	Mean (%)	SD	Min (%)	Max (%)	p-value
Proximal	44	63.3	16	29.4	86.5	0.33
Middle	37	57.8	19.8	18.7	85.9	
Distal	16	57.5	19.5	19.2	88.4	
Total	97	60.2	18.1	18.7	88.4	

SD, standard deviation.

The mean EF of the patients with absent, poor, good and excellent CACs was calculated as 50.4, 47, 60.5 and 70%, respectively. ANOVA showed a significant difference in the mean EF calculated for the different CAC grades in the patients (p < 0.001) (Table 4).

**Table 4:** Mean ejection fraction of patients in the different coronary collateral grades

Collateral grade	Sample size (n)	Mean (%)	SD	Min (%)	Max (%)	p-value
Absent	16	50.4	17.6	19.4	74.3	< 0.001
Poor	13	47	12	29.4	66.3	
Good	35	60.5	18.9	18.7	84.7	
Excellent	33	70	13.8	29.7	88.4	
Total	97	60.2	18.1	18.7	88.4	

SD, standard deviation.

A post hoc test was performed to determine the significance of the differences in mean EF calculated for each grade of CAC. There were significant differences between the mean EF calculated for patients with absent and excellent CACs (p = 0.004), and between the mean EF for poor and excellent CACs (p < 0.001). In addition, there was also a significant difference between the mean EF calculated for patients with poor and good CACs (p < 0.05).

The mean EF of the patients was also correlated with the CAC grades. In assessing the correlation between the mean EF and the CAC grades, a Spearman’s correlation analysis was performed. This revealed a positive correlation coefficient (r = 0.478) that was significant (p < 0.001) between the mean EF of the patients and the CAC grades. This showed that the patients with better CAC grade had a higher mean EF.

## Discussion

Coronary collateral arteries have their origin from the same embryonic precursor as the native coronary arteries during embryogenesis; therefore the foundation of these collateral arterial networks is laid down during embryonic life and is present in the newborn.^9^,^25^ The normal human heart contains interconnecting channels,^26^ hence, coronary collateral pathways are present in both normal and diseased hearts.^21^ These channels exist as microvessels whose function is not clear and is not demonstrable angiographically when coronary circulation is normal or mildly obstructed.^11^,^26^

Functional collaterals were suggested to have developed from hypertrophic evolution of the vessels present in the normal heart.^6^ This evolutionary process is triggered by myocardial ischaemia and/or an increase in the pressure gradient in the collateral network.^8^,^25^,^27^ Due to this pressure gradient, there is an increase in the volume of blood propelled through these channels. They progressively dilate and are eventually angiographically visible as coronary collateral channels.26 The pressure gradient also results in an increased fluid shear stress in the vessel.^28^ This fluid shear stress is a primary morphogenic physical factor that determines the size of the developing collateral vessel.^25^

In the present study, the best developed CACs were recorded in those patients who had proximally located lesions (40.9%). Excellent collaterals were found in 29.7 and 25% of middle and distally located lesions, respectively. The more proximally located the lesion, the higher the pressure gradient between the normal (collateral-donating) coronary artery and the obstructed (collateral-receiving) vessel. In addition, the more proximal the lesion was situated, the greater the mass of ‘at risk’ ischaemic myocardium.^29^ Therefore, the highest prevalence of excellent collaterals in patients with proximally located lesions in the present study may have resulted from the combination of these factors (increased pressure gradient and myocardial ischaemia). Consequently, this results in an increased stimulus for collateral vessel formation.

It is apparent from the literature reviewed that there are no reports on the relationship between the situation of the lesion and LV function. In the present study, the mean EF calculated for the patients with proximally located lesions was the highest (63.3%) compared to mean EF for the middle (57.8%) and distally (57.5%) located lesions (Table 3). However, the current study did not find any significant difference in the prevalence of CACs with regard to the location of atherosclerotic lesion and the resultant preservation of LV function.

There are conflicting reports with regard to the functional importance of coronary collateral arteries. Sheehan et al.^30^ examined global left ventricular ejection fraction (LVEF) in patients with acute myocardial infarction before treatment and at discharge. They reported that global LVEF increased in patients with CACs but was the same in patients without coronary collaterals.

Habib et al.31 divided patients who failed to canalise at 90 minutes after administration of a thrombolytic agent, into two groups (with and without collaterals) and reported that global LVEF was significantly greater in patients with CACs at hospital discharge. On the contrary, Wackers et al.^32^ found no difference in the global LVEF in patients with and without CACs.

There is yet another supposed negative effect of coronary collaterals, namely coronary ‘steal’. This occurs either when the pressure in the donor vessel is suddenly low or when there is higher resistance in the collateral pathway.^33^ Therefore, it results in the flow of blood from the region of the collateral-receiving vessel to the collateral-donating vessel. However, patients with poorly developed CACs are more prone to coronary steal than those with well-developed CACs.^33^

To our knowledge, this study is the first attempt at establishing a relationship between the different grades of CACs and LVEF in the presence of total coronary arterial obstruction. There was a significant difference (p < 0.001) in the mean EF calculated for the different grades of CACs. In addition, a post hoc test showed a significant difference in the mean EF between excellent and absent collaterals (p = 0.004) and excellent and poor collaterals (p < 0.001). Therefore the development of excellent collaterals has a significant supportive effect in the preservation of LV function compared to patients with absent or poor collateralisation.

There was also a significant positive correlation between CAC grades and mean EF calculated for the different CAC grades. Our study corroborated the findings of Sheehan et al.^30^ and Habib et al.,^31^that the presence of excellent and well-developed CACs had a significant role in the preservation of LV function.

In addition, the present study showed that, as the grades of the CACs increased, there was an improvement in the ability of these collaterals to preserve LV function. Consequently, LV myocardial perfusion was greater in patients with well-developed CACs where the native artery was totally occluded, and resulted in better preservation of LV function even in the face of an acute coronary event.^34^ To date, the significance of collateral circulation in coronary bypass surgery has not yet been fully investigated. However, it has been reported that the collateral circulation is favourable for the successful construction of coronary artery bypass grafts.^26^

From the result of this study, it can therefore be seen that the presence of well-developed CACs should be considered in decision making in the management of patients with coronary arterial obstruction. In the presence of an adequately preserved LV function by coronary collaterals in asymptomatic patients, a strong case can be made for no intervention. Anecdotally, most cardiac practitioners would be aware of patients with total coronary arterial obstruction who have been leading a normal life, and even engaging in high-intensity sport without symptoms. Therefore, the significance of the coronary collateral arteries should not be underestimated, as identification of the CACs is relevant in clinical decision making.^35^

The limitations to the current study include the absence of clinical records, which made it impossible to determine the patients with risk factors and co-morbid conditions, such as diabetes mellitus and hypertension, which may also have influenced collateral vessel development. This would have enhanced the study; however, the aim of this study was to evaluate the functional importance of coronary collaterals on LV function, which was achieved by analysing the angiographic records.

## Conclusion

The location of atherosclerotic lesion had no significant effect on the prevalence of CAC grades and the resultant LV function. However, with the development of well-functioning coronary collaterals, there was a significant improvement in the ability of these collaterals to preserve LV function.
